# Severe Gastroenteritis From Giardia lamblia and Salmonella Saintpaul Co-Infection Causing Acute Renal Failure

**DOI:** 10.7759/cureus.25288

**Published:** 2022-05-24

**Authors:** Sifullah Bashar, Avijit Das, Saliha Erdem, Wasif Hafeez, Rana Ismail

**Affiliations:** 1 Internal Medicine, Sinai-Grace Hospital, Detroit Medical Center, Wayne State University, Detroit, USA; 2 Internal medicine, Wayne State University, Detroit, USA; 3 Internal Medicine, Wayne State University School of Medicine, Detroit, USA; 4 Internal Medicine, Sinai Grace Hospital, Detroit Medical Center, Detroit, USA; 5 Clinical Research, Michigan State University School of Osteopathic Medicine, Detroit, USA

**Keywords:** co-infection, parasitic infection, non-typhoidal, salmonellosis, giardiasis

## Abstract

Giardiasis, a feco-oral route parasitic intestinal infection, and Salmonellosis, a foodborne enteric and extraintestinal bacterial infection, remain major public health issues in countries that lack adequate sanitation, safe water supply, and proper food handling. Here we report a case of *Giardia lamblia* and invasive non-typhoidal Salmonella disease co-infection in a patient with a history of recent travel to Mexico.

## Introduction

Millions of people who travel from temperate industrialized countries to the tropical regions of Asia, Africa, and Central and South America become susceptible to various infectious diseases that are endemic to these regions. Most affected individuals experience acute traveler's diarrhea caused by viral, bacterial, or protozoal entero-pathogens [1].

*Giardia lamblia* is the most common protozoal intestinal parasite worldwide [2], causing more than 300 million annual cases globally, and it is most prevalent in tropical and subtropical countries. This parasite infects humans and animals and sheds in their feces. It is highly transmissible through the fecal-oral route, from person to person (close contact with an infected person), and via contaminated food and water [3], often causing outbreaks in places lacking proper sanitation. Recently, *Giardia lamblia* has also become implicated in sexually transmitted diseases among homosexual men [4] and opportunistic infectious diseases among immunocompromised hosts [5]. According to estimates, 50% to 75% of infected individuals with *Giardia* are asymptomatic [6], while other affected individuals exhibit gastrointestinal symptoms and present with acute or chronic diarrhea, diffuse abdominal pain, and dysentery. Symptoms are self-limited and often resolve spontaneously, but some asymptomatic hosts become carriers and persistently excrete cysts for months or years [7].

*Salmonella* consists of Gram-negative facultative anaerobic bacilli bacteria with motile flagella and more than 2,500 serotypes [8]. As a noncoliform enteric bacteria, *Salmonella* infection in humans usually ensues from ingesting fecally contaminated water and animal food products, such as eggs, poultry, and meat, and causes mild-to-severe systemic salmonellosis or regional enteritis. Many *Salmonella* subspecies derived from the main species *Salmonella enterica* cause diarrheal illness; however, *Salmonella typhi*, *Salmonella paratyph*i A, B, and C, and *Salmonella sendi* are exclusively human pathogens, primarily inducing enteric fever rather than diarrhea (such as typhoid or paratyphoid fever). The outer surface layer consisting of polysaccharide capsules lends *Salmonella* its virulence and resistance to drugs. In the United States, *Salmonella* bacterial infection accounts for more than 1.3 million infections per year, while typhoid fever from *Salmonella typhi*, a severe and deadly infection, accounts for less than 500 cases per year, mainly acquired from recent international travel to endemic regions [9]. *Salmonella* symptoms of diarrhea, abdominal pain, or fever emerge within a few hours after exposure and may last up to a week. These symptoms may resolve independently without treatment, and only severe infections necessitate hospitalization and antibiotic therapy. Some infected people remain asymptomatic.

Both *Giardia* and *Salmonella* infections cause gastrointestinal symptoms characterized by diffuse abdominal pain, fever, vomiting, and watery diarrhea but rarely result in severe acute renal failure. We report a case of a returning traveler from Mexico presenting with co-infection of *Giardia lamblia* and *Salmonella* accompanied by an atypical manifestation of severe acute renal failure, successfully managed by aggressive fluid infusion without hemodialysis.

## Case presentation

A 73-year-old African American male with a history of left nephrectomy due to gunshot injury, hypertension, and type 2 diabetes presented to the emergency department (ED) with chief complaints of profuse vomiting, diarrhea, diffuse abdominal pain associated with fever, chills, and decreased urination. He denied any headache, vision change, chest pain, cough, rash, hematemesis, or melena. He had recently traveled to Mexico for vacation and returned home one day before his hospital presentation. On presentation, his vitals were remarkable for hypotension (blood pressure of 86/44 mmHg) and tachycardia (heart rate of 102 beats/minute), but with normal respiratory rate (16 breaths/minute) and spO_2_ of 97% on room air, weight of 131 kg, and no fever (37.7°C). He also exhibited signs of severe dehydration with poor skin turgor and sunken eyes, and had an acute abdomen, which was tender to palpation in all quadrants with normal bowel sounds. Other systemic examinations were unremarkable. He received a bolus of 3 liters of Ringer lactate and 3 liters of normal saline in the ED, which raised his blood pressure to 105/58 mmHg. The patient was started on IV metronidazole 500 mg four times daily in the ED along with aggressive rehydration with normal saline at 125 mL/hour. Initial laboratory testing was primarily unremarkable except for a slightly elevated lactic acid at 2.1 mmol/L (reference range: 0.4-2.0 mmol/L), low potassium at 3.1 mmol/L (reference range: 3.5-5.1 mmol/L), low chloride at 93 mmol/L (reference range: 98-107 mmol/L), and low bicarbonate at 19 mmol/L (reference range: 22-26 mmol/L). The labs also revealed an increase in blood urea nitrogen (BUN) at 45 mg/dL (reference range: 7-25 mg/dL), creatinine at 6.64 mg/dL (Table 1) (baseline 1.5 six years ago), and an elevated anion gap of 18 mmol/L (reference range: 8-12 mmol/L). Urinalysis and liver function tests were normal.

**Table 1:** Electrolytes, BUN, Cr, and WBC after admission BUN, blood urea nitrogen; Cr, creatinine; WBC, white blood cells

		Day 1	Day 2	Day 3	Day 6	Day 7	Day 8	Day 9	Day 11
Sodium (reference range: 136-145 mMol/L)	130	134	134	130	132	134	136	139	143
Potassium (reference range: 3.5-5.1 mMol/L)	3.1	2.8	2.8	3.0	3.4	3.4	3.5	3.8	3.8
Chloride (reference range: 98-107 mMol/L)	93	95	97	99	101	109	109	115	115
HCO_3_ (reference range: 21-31 mMol/L)	19	24	18	18	15	15	15	16	20
BUN (reference range: 7-25 mg/dL)	45	45	45	60	68	63	52	35	14
Cr (reference range: 0.70-1.30 mg/dL)	6.64	6.93	7.45	8.66	9.88	8.31	5.08	2.64	1.42
Phosphate (reference range: 2.5-4.5 mg/dL)			4.9	4.4	4.8	4.6	3.5	2.8	
Magnesium (reference range: 1.6-3.0 mg/dL)	1.6		1.6	2.0	2.1	1.9	1.8	1.5	1.8
WBC (reference range: 3.5-10.5 K/cumm)	5.6	4.1	3.8	5.7	7.3	6.6	8.0	8.4	5.7

**Figure 1:**
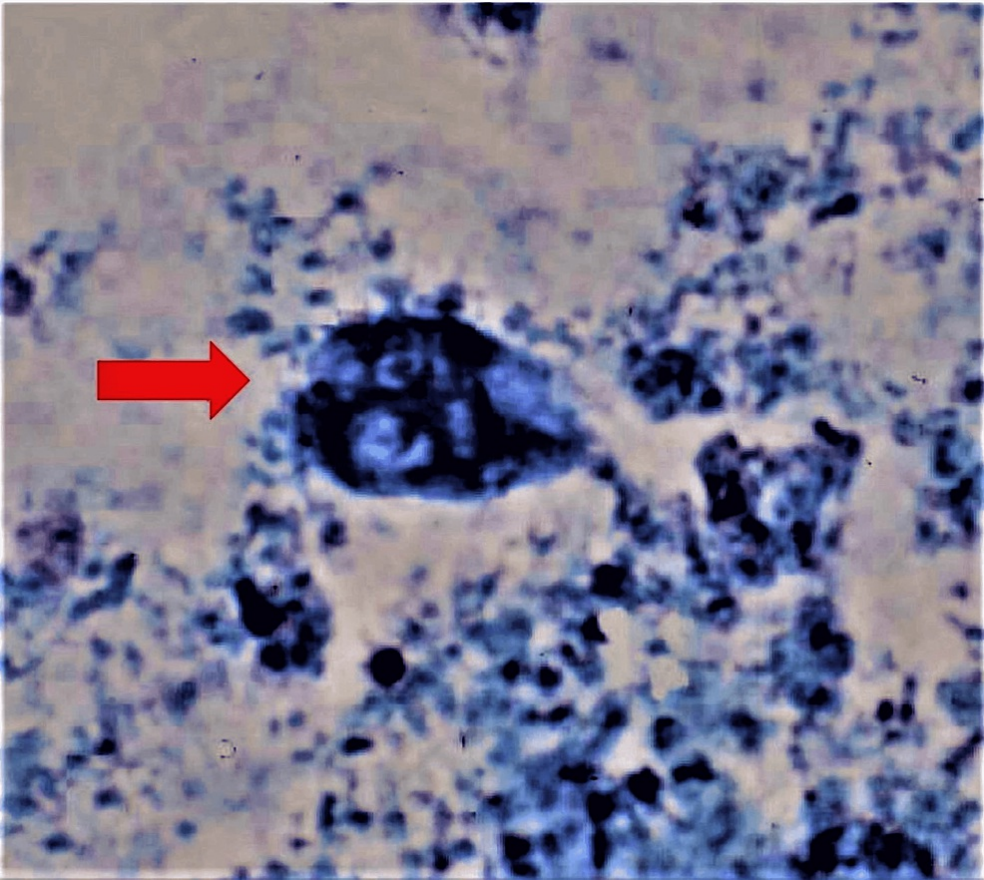
Giardia trophozoite under light microscopy (methylene blue stain)

**Figure 2:**
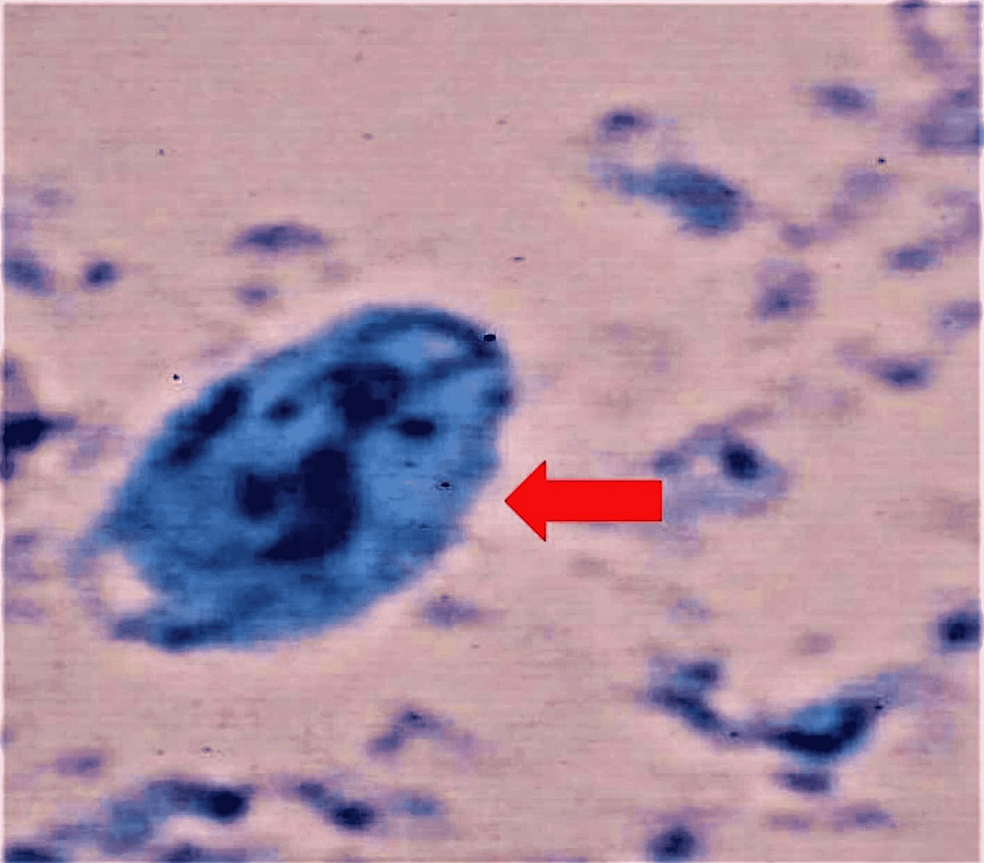
Giardia cyst under light microscopy (methylene blue stain)

The stool studies were positive for *Giardia lamblia* (Figures 1, 2), and subsequent stool culture showed *Salmonella* species *Saintpaul*. Further testing on *Clostridium difficile*, ova and parasites, and fecal occult blood were negative. His initial CT of the abdomen with contrast revealed no evidence of appendicitis or bowel obstruction. The patient was hypokalemic and replaced to establish potassium replenishment. A Foley catheter was inserted to monitor strict input and output.

On day 2 of admission, the patient reported that he had few bowel movements. However, his creatinine trended upward despite aggressive hydration and reached 7.45 mmol/L (Table 1). The normal saline infusion rate was further increased from 125 to 150 mL/hour to achieve better rehydration, and the patient’s 24-hour urine output measured 450 mL. The anion gap remained elevated at 19 mEq/L. On day 3, the patient’s blood cultures were positive and grew *Salmonella* species *Saintpaul*, for which we started him on IV ceftriaxone 2 gm every 24 hours. His creatinine continued to trend upward at 9.42 mmol/L (Table 1) despite the continuous aggressive hydration, but his anion gap dropped to 13 mmol/L, within the normal range. On day 4, his creatinine elevation peaked during his hospital course and reached 9.88 mmol/L (Table 1); as a result, the normal saline infusion rate increased to 200 mL/hour. His creatinine dropped to 8.31 mmol/L (Table 1) the following day, and it continued to trend down in response to aggressive hydration. With a steady downtrend in creatinine levels, we readjusted the infusion rate with saline to 125 mL/hour for the rest of the hospital course after achieving a creatinine level of 2.64 mmol/L (Table 1). On day 8, repeat blood cultures showed negative growth for initial *Salmonella*, and treatment with IV ceftriaxone switched to oral ciprofloxacin 500 mg once daily. The patient was clinically stable and was discharged on day 9 with a creatinine level of 1.42 mmol/L (Table 1). We recommended he continue oral ciprofloxacin for five days for a total treatment duration of 14 days and follow up with his primary care physician within one week at an outside hospital system.

## Discussion

The rapid rise in international travel from the United States to developing countries has increased the prevalence and emergence of travel-related infectious diseases caused by uncommon pathogens. Mostly, acute infectious diarrhea is caused by enteric bacteria, viruses, or parasitic organisms from contaminated water and foodborne sources [1], and is often responsible for considerable morbidity and mortality if left untreated. Although *Giardia* and *Salmonella* infections are highly endemic in countries with suboptimal sanitation and unsafe water supplies, they are relatively rare in the United States and are commonly imported by returning international travelers.

Giardiasis, the waterborne parasitic infection, could display an inconsistent clinical course that ranges from a self-limited asymptomatic presentation to symptomatic or persistent acute or chronic diarrheal disease [10]. A positive *Giardia* antigen test based on detecting microscopic cysts or trophozoites in the stool specimen usually confirms, with high sensitivity, the diagnosis of *Giardia lamblia*. Although there are more modern diagnostic techniques for giardiasis, such as immunoassays or molecular tests, the traditional and cost-effective microscopic test remains the gold standard. *Giardia lamblia* often produces non-life-threatening gastrointestinal distress and diarrhea [2,5], but it is very unusual for *Giardia* infection alone to cause severe diffuse abdominal pain, fever, and/or dysentery [2]. Giardiasis is frequently perceived as a benign disease because, in most instances, symptoms resolve spontaneously. However, the possibility of a co-infection with other pathogens should always be investigated in the presence of severe sepsis. Our patient presented with typical giardiasis symptoms of vomiting, diarrhea, and diffuse abdominal pain, but he also experienced an atypical acute renal failure. Many people with *Giardia lamblia* have either achlorhydria or hypochlorhydria [11]. Moreover, patients with abnormally low hemoglobin or those who undergo gastric surgery often present with some *Giardia lamblia* infections [5] but without a definitive cause-effect relationship. Since our patient was previously healthy, had never received H2 blockers or proton pump inhibitors, and had never undergone gastric surgery, it is unlikely to attribute his severe illness to hypochlorhydria alone.

On the other hand, *Salmonella* infections can be typhoidal or non-typhoidal (NTS); the latter could be invasive (iNTS) or non-invasive. Some factors that increase the susceptibility to acquiring *Salmonella* infection include any condition that decreases stomach acidity or intestinal integrity, inflammatory bowel diseases, cytotoxic chemotherapy, prior gastrointestinal surgery, or alteration of the intestinal microbiome by antibiotic administration. Most cases of NTS present with self-limiting gastroenteritis in immunocompetent persons, and symptoms start 12 to 48 hours after exposure, ranging from nausea, cramping, and abdominal pain, to fever, vomiting, and diarrhea. Nevertheless, iNTS is highly endemic and overburdening in some geographical areas such as sub-Saharan Africa and, to a lesser degree, Europe [12]; however, it is also prevalent in Asia and Latin America but possibly underreported due to lower testing and surveillance. Invasive NTS is frequently associated with extremes of age, malnutrition, HIV infections, clinical malaria, and sickle cell disease [13]. Although iNTS often manifests as a nonspecific febrile illness with hepatosplenomegaly and respiratory symptoms, it is not typically associated with diarrhea. The hallmark of iNTS lies in causing bloodstream infection (bacteremia or sepsis), sterile site infections, and sometimes focal infections [14]. Some iNTS serovars cause life-threatening bloodstream infections if left untreated, especially among the immunocompromised [12]. One international study showed a 25% case fatality rate, as reported among hospitalized bacteremic patients [14]. Rare complications of iNTS could include disseminated infections such as meningitis, muttering delirium, pancreatitis, endocarditis, severe pneumonia, pericarditis, hemolytic uremic syndrome, arthritis, and osteomyelitis. Our patient had *Salmonella Saintpaul* bacteremia that caused iNTS; we postulate that the simultaneous infection with *Giardia* enabled and promoted *Salmonella*'s invasion of the intestinal tissue and mucosa and caused extraintestinal bacteremia and iNTS.

Once *Giardia*'s cyst is ingested, trophozoites emerge in the small intestine, and instead of directly invading the tissue, they use their sucker plates to attach themselves to the intestinal villi [2,7]. The consequent damage to the intestinal mucosa and the shortening of brush-border microvilli with or without villous atrophy, along with disaccharidase deficiency, induce a host immune response and increase intestinal permeability with anion hypersecretion and fluid accumulation in the intestine. Simultaneously, the damage extends to modify the bacterial flora, which may stimulate protozoal pathogenicity and intestinal barrier dysfunction and induce apoptosis of enterocytes [15].

The literature has shown that acute renal injury (AKI) or acute renal failure could be associated with *Salmonella* infection; for instance, AKI has occurred in 2.5% of patients with typhoid fever [16]. In the setting of *Salmonella* infection, AKI is likely due to mixed prerenal and intrarenal etiologies, such as the renal hypoperfusion due to severe vomiting and diarrhea and the toxic damage induced by *Salmonella* septicemia. Aggressive treatment with IV hydration with crystalloids and prompt initiation of proper antibiotics can prevent any kidney injury that may require hemodialysis. Cases of *Giardia lamblia* and *Salmonella* co-infections with severe sepsis leading to end-organ damage were reported outside the USA in Egypt, Japan, and other sub-Saharan African countries but are infrequent in the USA [17].

## Conclusions

This case highlights the importance of taking a careful travel history and identifying bacterial co-infection with parasites when a patient presents with severe illness. Appropriate and timely antibacterial management based on cultures is necessary to treat a possible life-threatening iNTS. Aggressive hydration and close monitoring of renal function are required to reverse renal injury and prevent renal failure and the subsequent long-term hemodialysis requirements.
